# Vascular Damage in Kidney Disease: Beyond Hypertension

**DOI:** 10.4061/2011/232683

**Published:** 2011-08-14

**Authors:** Andréa E. M. Stinghen, Roberto Pecoits-Filho

**Affiliations:** ^1^Basic Pathology Department, Laboratory of Experimental Nephrology, Universidade Federal do Paraná, 80531-980 Curitiba, PR, Brazil; ^2^Center for Health and Biological Sciences, Pontifícia Universidade Católica do Paraná, Rua Imaculada Conceição, 1155, 80215-901 Curitiba, PR, Brazil

## Abstract

Chronic kidney disease (CKD) is highly prevalent and a multiplier of cardiovascular disease (CVD) and cannot be completely explained by traditional Framinghan risk factors. Consequently, greater emphasis has been placed in nontraditional risk factors, such as inflammation, endothelial dysfunction, sympathetic overactivation, protein-energy wasting oxidative stress, vascular calcification, and volume overload. The accumulation of uremic toxins (and the involvement of genetic factors) is responsible for many of the clinical consequences of a condition known as uremia. In this brief paper, we discuss mechanisms involved in the vascular damage of CKD patients, aiming to point out that important factors beyond hypertension are largely responsible for endothelial activation and increased CVD risk, with potential impact on risk stratification and development of novel therapeutic options.

## 1. Introduction

Chronic kidney disease (CKD) is a disease in exponential growth, and, along with other chronic diseases, is responsible for 25 million deaths per year, mainly due to the multiplication of cardiovascular disease (CVD) [1]. Cardiovascular disease is independently associated with CKD, and this cardiovascular risk cannot be completely explained by traditional Framinghan risk factors (age, lifestyle, left ventricular hypertrophy, dyslipidemia, hypertension, and diabetes mellitus) [2]. Consequently, greater emphasis has been placed in nontraditional risk factors, such as inflammation, endothelial dysfunction, sympathetic overactivation, protein-energy wasting oxidative stress, vascular calcification, and volume overload [3, 4]. In patients with stage 5 CKD, CVD risk is five times greater than in the general population, even after stratification for age, sex, race, and diabetes [5]. Hypertension is very common in this population, and its prevalence increases with progression of the disease. Moreover, with retention of sodium, expansion of extracellular volume, and activation of renin angiotensin system, all highly prevalent in CKD, hypertension becomes refractory to treatment and an universal condition in advanced stages of CKD. However, hypertension alone, although important and highly prevalent, cannot by itself justify the enormous cardiovascular burden in CKD.

In the progression of CKD, the kidneys lose their ability to effectively remove toxic compounds from the bloodstream for subsequent formation of urine, resulting in its accumulation in the body. The accumulation of these uremic toxins (and the involvement of genetic factors) is responsible for many of the clinical consequences of a condition known as uremia [6, 7]. Among the toxic effects of uremia, the most important due to its impact on clinical outcome is the multiplication in cardiovascular risk. In this brief paper, we discuss mechanisms involved in the vascular damage of CKD patients, aiming to point out that important factors beyond hypertension are largely responsible for endothelial activation and increased CVD risk, with potential impact on risk stratification and development of novel therapeutic options.

## 2. Chronic Kidney Disease and Uremic Toxicity

Currently there are over 100 identified uremic compounds, which are classified according to their physical and chemical characteristics of removal by dialysis in: (i) small water-soluble compounds (e.g., urea and uric acid), which are easily removed by dialysis and not necessarily with a functional toxicity; (ii) compounds of moderate molecular weight (e.g., *β*2-microglobulin and leptin), only removed by special dialysis strategies and that affect many organ systems; (iii) compounds bound to proteins, generally with low molecular weight (e.g., phenols and indoles), difficult to remove by dialysis and capable to enhance many toxic activities in the body [8–10]. Uremic toxins cannot be defined simply as substances present in body fluids of uremic patients. It should also be shown as connection between the toxic substance and one or more events, pathobiological or clinical [6]. Uremic toxicity affects almost every organ system; therefore, cardiovascular damage is known to have the major impact on morbimortality of CKD patients [8].

## 3. Endothelial Dysfunction and CKD

The endothelium is constituted by a single layer of cells at the interior surface of blood vessels, and it is the largest organ in the body and the main regulator of vascular homeostasis, covering a surface area of approximately 4.000 to 7.000 m^2^ [11]. Moreover, because of its enormous surface area, the endothelium is involved in many disease conditions, but mostly it is the victim of the so-called cardiovascular risk factors, for example, hypertension and diabetes [12]. Endothelial dysfunction is a common event described in renal failure, both chronic and acute, as well as in all end-stage renal diseases. It is believed that the accumulation of uremic toxins may lead to an endothelial inflammatory response [9, 13]. Indeed, exposure of endothelium to these toxins (a manifestation of uremic toxicity) leads to changes in cellular phenotype and production of many proinflammatory molecules [14, 15], with rapid degeneration of cardiovascular system. 

In the progression and development of atherosclerosis, some important toxins can be mentioned. First, the production of reactive oxygen species (ROS) in endothelial cells, which is stimulated by indoxyl sulfate, may contribute to the inflammatory state found in CVD [16]. The interaction of ROS with proteins can be responsible for the expression of nitrotyrosine by endothelial cells and high-level expression of receptors for advanced glycation end products (AGEs) [17–19]. In the cardiovascular system, AGEs accumulation contributes to stiffening of the arteries due to its binding to collagen and elastin in a disorderly and not functional way [20]. In addition, uremic toxicity leads to an impairment in endothelium nitric oxide (NO) synthesis, which plays a crucial role in vascular protection since NO inhibits proliferation and migration of VSMC, expression of adhesion molecules, and platelet aggregation [21]. 

Second, symmetric dimethylarginine (ADMA) is an important protein-bound, low-molecular-weight potential uremic toxin and reported to be a novel risk factor for endothelial dysfunction, strongly correlated with impaired flow-mediated vasodilation and with carotid intima-media thickness [6, 22, 23]. Moreover, high oxidative stress, AGEs, and ADMA decrease the endogenous nitric oxide synthase (eNO) activity and cause impairment in endothelium NO availability. NO pathway may be additionally inhibited indirectly by endothelial microparticles (EMPs), small vesicular fragments of the endothelial cell membrane released during activation or apoptosis [19]. EMPs possible can be induced by indoxyl sulfate and p-cresyl sulfate (both classified as small molecules), and, together with AGES and ADMA, are compounds with the potential to induce vascular damage [24]. 

In addition, parathyroid hormone (PTH) is also recognized as a major uremic toxin, although its increased concentration in CKD patients is merely attributable to enhanced glandular secretion, rather than to decreased removal by the kidneys. Excess PTH gives rise to an increase in intracellular calcium, which results in functional disturbances in most organ systems [25]. Later in the progression of vascular disease development, vascular calcification is markedly higher in CKD patients. Hyperphosphatemia is a driving force in the pathogenesis of vascular calcification (VC) and secondary hyperparathyroidism associated with renal failure. Because secondary hyperparathyroidism is associated with morbidity and mortality in patients with chronic kidney disease, suppression of parathyroid hormone (PTH) and minimization of associated derangements in mineral metabolism are cardinal therapeutic goals [26]. Indeed, Neves et al. [27] demonstrated in an animal model of uremia that infusion of synthetic PTH at a supraphysiologic rate contributed to vascular calcification. 

Recently, we demonstrated using *in vitro* and *in vivo* models that exposure of endothelium to uremic plasma results in time- and CKD-stage-dependent increased expressions of monocyte chemoattractant protein-1 (MCP-1), soluble vascular adhesion molecule-1 (sVCAM-1), and interleukin-8, which suggest a link between vascular activation, systemic inflammation, and uremic toxicity [28]. Furthermore, we investigate the effect of a toxin called endotoxin (ET) that may interfere in endothelial dysfunction and is commonly observed in CKD patients. ET is constituted by a lipopolysaccharide (LPS) complex present in the outer membrane of gram negative bacteria. In this group of patients, the sources of these molecules could be contamination of tissues, fluids, or foreign bodies and translocation from the intestinal lumen related to an impaired intestinal barrier function. There is increasing evidence that patients with CKD develop signs of fluid overload in the early phases of the disease, and this may be a stimulus for inflammatory activation [29] that may lead to accelerated CVD and rapid progression of renal failure [30, 31]. One of the main epiphenomena of endothelial dysfunction is the decline of eNOS. Patients in CKD stage 5 have decreased NO production which in turns causes an impaired endothelium-dependent vasodilatation. Furthermore, decline production in NO also can lead to elevated levels of ET and proinflammatory cytokines found in uremia resulting in attenuated prothrombotic potential of endothelial cells [32].

## 4. Genetic Contribution in CVD in CKD Patients

Epigenetics is a new area dedicated to the study of the changes that occur in primary cell genome as a result of changes in the conformation of DNA generated by the environment. These may include cytosine methylation, histone modifications, chromatin remodeling, and silencing RNA-dependent [33]. Changes in DNA through methylation (addition of methyl groups at carbon 5 of cytosine of CpG nucleotide groups) have important regulatory role in pathological processes and contribute to normal development and differentiation of tissues. Epigenetic modifications are crucial in several diseases such as cancer, atherosclerosis, and autoimmune diseases [34], and recently some studies have demonstrated that gene inactivation of estrogen receptor-*α* has an important role in atherogenesis and aging of the vascular system [3]. Previous studies have shown that dyslipidemia, oxidative stress, hyperhomocysteinemia, and inflammation may result in imbalance in DNA methylation, so it can be suggested that the uremic environment may also have a significant effect on the epigenome [33], as demonstrated by Stenvinkel et al. in studies performed in peripheral blood lymphocytes from hemodialysis patients, where they found signs of DNA hypomethylation [34]. Homocysteine and uremic toxin and its precursor, S-adenosylhomocysteine, are elevated in uremia [35]. Likewise, recent studies have demonstrated *in vitro* that homocysteine causes methylation of estrogen receptor-*α* genes and promoter ApoE gene (considered antiatherosclerotic) [36, 37].

Zaza et al. demonstrated in peripheral polymorphonuclear cells from CKD patients through genomic studies involving more than 15,000 candidate genes that *MIF*, *CXCL12,* and *IL8RB* genes were independently associated with inflammation, and *CXCL12* and *IL8RB* genes were inversely correlated to C reactive protein (CRP) and highly expressed in peritoneal dialysis patients and CKD, respectively [38]. The Stroma cell-derived factor 1 (SDF-1), also known as CXCL12, expressed by *CXCL12* gene is a CXC chemokine [39] and exerts its activity by binding its receptor, a G protein called CXCR4 on the cell surface [40]. SDF-1 was originally characterized by stimulating T lymphocytes, B lymphocytes, and monocytes. However, it was discovered that his action is restricted not only in immune and hematopoietic cells but also in the central nervous system and endothelial cells [41], developing important roles in pathophysiology processes such as inflammation, angiogenesis, and wound healing. Furthermore, SDF-1 is critical for growth, survival, and metastatic spread of various tumor types [40]. Additionally, it was suggested that SDF-1 plays a pivotal role in induced stem cell mobilization, and targeted expression of SDF-1 after myocardial infarction was shown to result in increased engraftment of bone-marrow-derived stem cells into infarcted myocardium [42]. In this context, Jie et al. showed an increase of this cytokine in pre-dialysis patients samples when compared with controls [43]. 

Despite the constant uremic state, differences like socioeconomic and cardiovascular risk and risk factors as hypertension and diabetes in CKD patients are known, pointing that additional factors should be involved, like genetic risk factors [44]. It was demonstrated in biopsies from skeletal muscle from hemodialysis patients, genes that were upregulated (*GADD45A*, *BTG2*, *PDE4B*, and *CEBPD*), and a gene (*TOB1*) that was downregulated compared with controls [45]. Also, recently, it was demonstrated in a 67,093 study population from 20 predominantly population-based cohorts within the CKDGen consortium, a new susceptibility loci for reduced renal function. They identified 20 new replicated loci associated with GFR and CKD. Of these, 13 are likely to be involved in renal function and in susceptibility to CKD, whereas 7 are likely to be associated with creatinine production or secretion, identifying common genetic variants in genes related to nephrogenesis (*ALMS1*, *VEGFA, *and, potentially, *DACH1*), glomerular filtration barrier formation and podocyte function (*DAB2*, *PARD3B, *and *VEGFA*), angiogenesis (*VEGFA*), solute transport (*SLC7A9*, *SLC34A1*), and metabolic functions of the kidney (*PRKAG2 *and, potentially, *GCKR *and *LASS2*) [44]. These recent technological advances in discovering new disease susceptibility genes will improve the understanding of important mechanisms for renal development and CKD pathogenesis, contributing to establish novel treatment strategies based on genomic information related to kidney disease.

## 5. Conclusions

Beyond hypertension, novel mechanisms involved in the vascular damage in CKD have been recently described. Particularly, the involvement of uremic toxins and epigenetics on the uremia-related CVD emerge as important candidates responsible for endothelial activation and increased CVD risk, with potential impact on risk stratification and development of novel therapeutic options.
